# Responding to vulnerable patients with multimorbidity: an interprofessional team approach

**DOI:** 10.1186/s12875-022-01670-6

**Published:** 2022-03-30

**Authors:** Judith B. Brown, Sonja M. Reichert, Pauline Boeckxstaens, Moira Stewart, Martin Fortin

**Affiliations:** 1 Centre for Studies in Family Medicine, Western Centre for Public Health and Family Medicine, 2nd floor, Schulich School of Medicine & Dentistry, Western University, 1151 Richmond St. London, London, ON N6A 3K7 Canada; 2 Faculty of Medicine and Health Sciences, Department of Public Health and Primary Care, Ghent University, C. Heymanslaan 10, ingang 42 – verdieping, 5 9000 Ghent, Belgium; 3 Unité de Médecine de Famille, CIUSS du Saguenay-Lac St-Jean, 305 St-Vallier, Chicoutimi, Québec G7H 5H6 Canada

**Keywords:** Multimorbidity, Vulnerable patients, Interprofessional teams, Primary care, Qualitative

## Abstract

**Background:**

People with multimorbidity, who may be more vulnerable to certain social determinants of health, often require care by an interprofessional primary healthcare (PHC) team that can tailor their approach to address the multiple and complex needs of this population. This paper describes how the needs of vulnerable patients experiencing multimorbidity are identified and provided care by innovative interprofessional PHC teams during an innovative one-hour consultation, outside of usual care.

**Methods:**

This was a descriptive qualitative study. Forty-eight interviews were conducted with 20 allied healthcare professionals: (e.g., social work, pharmacy); 19 physicians (e.g., psychiatry, internal medicine, family medicine); and 9 decision makers. The thematic analysis was iterative using an individual and team approach to identify the main themes and exemplar quotations for illustration.

**Results:**

Participants described patients with multimorbidity who were vulnerable as those experiencing major challenges accessing and navigating the healthcare system. Mental health issues were a major contributor to being vulnerable and often linked to common social determinants of health. Cultural factors were identified as potentially causing patients to be vulnerable. Participants articulated how the collaborative nature of the team generated new ideas and facilitated creative recommendations designed to meet the specific needs of each patient.

**Conclusions:**

This one-time consultation went beyond the assessment of a patient’s multimorbidity by including a psycho-social-contextual understanding of vulnerability within the healthcare system. Findings may have important clinical and policy implications in the adoption and implementation of this approach and further assist vulnerable patients with multimorbidity in having their complex needs addressed.

## Introduction

The rising prevalence of chronic conditions comes with a rise in multimorbidity. A systematic review on 45 studies conducted from 2007 to 2017 showed that the overall prevalence of multimorbidity was 66.1% (with multimorbidity defined as having ≥ 2 chronic conditions), and 44.2% (with multimorbidity defined as having ≥ 3 chronic conditions) in older adults in high-income countries [1].

People who have multimorbidity may be at higher risk of being vulnerable. The concept of vulnerability in the health literature acknowledges the connection between healthcare disparity and multiple aspects of vulnerability such poverty and mental health [2]. Vulnerability has been linked to a phenomenon previously described as the inverse care law – wherein those patients demonstrating the greatest healthcare needs receive the least healthcare [3, 4]. Often the inverse care law is partially attributed to a lack of health insurance such as in the United States. However, it holds true in countries with universal health insurance systems such as Canada and the United Kingdom as well [4–7]. For example, in deprived areas multimorbidity occurs more often and at an earlier age as noted by researchers in the United Kingdom [8].

As physical morbidities increase in number so does the prevalence of mental illness; furthermore, mental illness has been found to be substantially greater for those patients in socioeconomically deprived areas [8]. It is difficult to determine if some of the psychological distress experienced by patients with multimorbidity is related to their disease severity as reported in the literature [9] or if patients had premorbid mental health issues prior to the onset of their chronic conditions. Nonetheless, psychological distress and mental health issues contribute to their vulnerability [9].

Patients who are vulnerable are more likely to face healthcare disparities and fragmentation of care such as difficulties in seeing a specialist or seeing multiple specialists [10–12], receiving less aggressive treatment for cancer and greater barriers accessing quality primary care [13]. Patients with multimorbidity who are also vulnerable may present additional challenges for clinicians who are already feeling overwhelmed and ill-equipped to manage the health needs of these vulnerable patients [14]. Studies have reported that physicians tend to be more directive with vulnerable patients, spend less time with them and provide less information regarding treatment options [14–16]. Research examining healthcare providers' perspectives towards caring for vulnerable patients suggests that some feel inadequately trained, question both how and why they should care for this population and lack information on relevant community resources [13, 16, 17]. Aside from their own prejudicial judgements, family physicians have also expressed their frustration with the disorganization and fragmentation of the healthcare system, which presents multiple barriers in serving patients with multimorbidity [13, 16, 18]. In contrast, other studies have focused on family physicians' empathy and empowerment and high levels of creativity and commitment in searching for individual solutions for family practice patients with multimorbidity who are vulnerable [15, 19].

Prior research has focused on training medical students, family physicians and other healthcare providers in building and sustaining therapeutic alliances as well as adapting practice to their vulnerable patients' needs and priorities [14, 17, 20, 21]. However, less research has focused on interprofessional collaborative approaches to elucidate relevant strategies to be used by teams in caring for vulnerable populations with multimorbidity.

To address these gaps, we examined the Telemedicine Interprofessional Model of Practice for Aging and Complex Treatments Plus (TIP Program), which is a patient-centered model of care for chronic disease management. Prior to the actual TIP consultation with the interprofessional team an initial assessment is conducted by a dedicated TIP registered nurse who meets with the patient and their primary care physician to determine the key issues to be addressed. Key goals of the TIP Program are to provide patient centered care, improve quality of care, help enable patient self-management, and introduce care coordination. This is done by a videoconference or in-person interprofessional case consultation where the patient and their primary care provider meet with a team of healthcare professionals to discuss health related issues and create a personalized patient care plan. At the end of the meeting the team, together with the patient, create a coordinated care plan with recommendations that identify current patient goals, follow-up plans, and identification of local resources [22, 23]. The work of Pariser et al. found high levels of satisfaction with the TIP intervention expressed by primary care physicians, patients and interprofessional team members [22]. Stewart and colleagues reported that the TIP intervention as studied in the RCT demonstrated improvement only for patients with a higher income while the qualitative component of this mixed method study suggested that an increase in patients' sense of hopefulness was linked to increased functional ability as a result of the TIP intervention [23]. Of note, neither of Pariser or Stewart focused specifically on how the TIP Program can address the needs of patients with multimorbidity who are vulnerable. This raises the question of what less tangible outcomes may apply to more vulnerable populations.

This paper reports one of the qualitative components of a mixed methods program called the Patient-Centred Innovations for Persons with Multimorbidity (PACE in MM) project. This paper examines how interprofessional PHC team members who provide the TIP program describe vulnerability in this context and address the needs of vulnerable patients with multimorbidity.

## Methods

This study used a qualitative descriptive approach to understand how an innovative interprofessional team consultation addressed the needs of patients with multimorbidity who are vulnerable [24]. A qualitative descriptive approach encourages the researcher to examine and describe the where, what and who and how of an event or experience [25, 26] and was deemed an appropriate methodology in this mixed methods program of research [23, 27].

### Setting

Four TIP Programs in Toronto, Canada providing a team consultation to patients with multimorbidity.

### Participants

Participants were initially recruited by the TIP Program Coordinator and the Program Leads who had developed TIP. Allied healthcare providers (e.g., social work, nursing, pharmacy), and physicians (e.g., family medicine, internal medicine, psychiatry) currently participating at one of the four main TIP Program sites were selected to reflect the team composition at each site. Family Physicians who had referred their patients to the Program, were approached by the Program Coordinator. Decision makers were defined as individuals who had a connection to the TIP Program, either at a managerial, municipal or provincial level, and were recruited by the TIP Program Leads. Contact with all participants was made either by phone or email. Subsequently, their contact information was sent to the study’s Research Assistant who organized the time and date of the interview. Informed consent was received from each participant before the interview began. Confidentially was assured.

### Data collection

Semi-structured individual interviews were conducted with each participant at their work location by three of the female researchers (JBB,SMR,PB). Some interview questions were generic for each group of participants such as their length of involvement with the Program, while other questions and prompts were tailored to their specific involvement in the Program (e.g., healthcare provider, decision maker). The interview guide included questions on the facilitators and barriers to performing the TIP Program activities, impact of the Program on patients, their family and primary care physician and teamwork. Participants were asked to describe how the TIP Program was perceived to address the needs of patients with multimorbidity who were vulnerable. A working definition of vulnerability was not utilized allowing an exploration of the participants' own description of vulnerability. The interviews, lasting from 30–60 min in duration, were audiotaped and transcribed verbatim by a professional transcription service. NVivo 10 software was used in the organization of the data.

### Data analysis

The data collection and analysis occurred concurrently reflecting an iterative approach. The thematic analysis was inductive [26, 28]. During the initial step of the analysis, three of the researchers (JBB,SMR,PB) independently reviewed and coded each transcript to generate all potential themes present in the data. They then met to examine their independent coding, which culminated in a consensus that informed the development of the coding template. This process analysis continued until all the transcribed interviews had been analyzed. Next the research team reviewed the main themes and sub-themes that had been inputted into NVivo. This step in the analysis facilitated the generation of the overarching themes and accompanying exemplar quotes. By the final stage of the analysis the researchers determined they had acquired sufficient data to understand and provide a fulsome description of the themes. As such no further interviews were conducted [29, 30]. The analysis for this paper specifically focused on understanding the participants' experiences in caring for patients with multimorbidity who are vulnerable.

### Trustworthiness and credibility

The trustworthiness and credibility of the analysis were ensured by using field notes following each interview, audio recording and verbatim transcripts, and independent and team analysis. Careful attention to how the researchers’ professional backgrounds (i.e., social work, family medicine), could influence the findings were taken into consideration The process of reflexivity was continuous throughout the study from the formulation of the research questions to the completion of the final manuscript [31–33]. As an example during analyses meetings the team would pause and acknowledge how their own perspectives and biases could be influencing the coding and generation of the themes.

### Final sample

The sample consisted of 48 participants. There were 20 allied healthcare providers (AHP) (e.g., nurses, social workers, pharmacists and dieticians) and 10 physicians (MD) (e.g., psychiatrists, general internists, and family physicians) who were part of the TIP team; 9 decision makers (DM); and 9 family physicians who had referred a patient to the Program (RFP). The average age was 46 years (range 23–70 years). There were 15 male and 33 female participants with an average years in practice of 20 years (range 2–46 years). Participants' length of involvement with the Program ranged from 4 months to 9 years and they had been affiliated with one specific TIP site interprofessional team.

### Ethics approval

This study was approved by the Health Sciences Research Ethics Board of The University of Western Ontario (#106,921) and all the methods were performed in accordance with the relevant guidelines and regulations of the Health Sciences Research Ethics Board of The University of Western Ontario. Informed consent was received from each participant before the interview began. Confidentially was assured.

## Findings

Understanding participants’ experiences of how the TIP Program addresses the needs of patients who have multimorbidity and are vulnerable was reflected by two overarching themes. First, their perceptions of the reasons certain patients with multimorbidity are vulnerable. Second, were the approaches participants used in caring for these vulnerable patients with multimorbidity which included: recognizing the social determinants of health; maximizing professional team collaboration; demonstrating cultural sensitivity, compassion and advocacy.

### The reasons some patients with multimorbidity are vulnerable

During the initial assessment by the TIP nurse and in the TIP consultation a thorough history is obtained including the patient's risk of being vulnerable. Overall, participants described patients with multimorbidity referred to the TIP Program as those who typically fall through the cracks: “*I think they fall through the cracks because they are overwhelmed. They have a low health literacy. You can give them the information but they don’t understand”.* (MD10). They were patients who also face major challenges with accessing the healthcare system: “*People who have the hardest time in accessing care.”* (HCP14), and furthermore vulnerable patients with multimorbidity were described as being unsure about how to navigate the healthcare system, “*They don’t know how to pound the pavement and figure out the system. We are here for them.”* (DM06). Participants reported that patients receiving the TIP intervention had over three chronic conditions which included, for example: arthritis, depression or anxiety, hypertension, chronic musculoskeletal problems and diabetes.

Mental health issues were identified as a major contributor to patients with multimorbidity being vulnerable: “*Consistently across the board is mental health.”* (DM05). Many family physicians referring patients to the TIP Programs observed that: *“The patients usually have a big psychiatric overlay.”* (RFP07). A specialist provider explained: “*Medicine is often the easiest part of the job. It’s all of the social context and quite often the psycho-social context that makes all of this stuff challenging.”* (MD09). Participants viewed mental health issues as often linked to the common social determinants of health such as the *“extremely poor”* (RFP02); *“homeless”* (DM03); or *“the immigrant population”* (DM04).

Cultural issues including language, different values and beliefs were identified as potential aspects of patients with multimorbidity being vulnerable. “*If their culture or their habits were different or they didn’t have money to implement some of these lifestyle things, I could see how that could be difficult.”* (RFP08)*.* A specific group noted as being vulnerable were seniors who were socially isolated. “*And we have a fairly large percentage of seniors that don’t have any family members. So that are just struggling on their own.”* (DM03)*.*

### Approaches used in caring for these vulnerable patients

In caring for these vulnerable patients with multimorbidity the Program used specific approaches including recognizing the social determinants of health, maximizing a collaborative interprofessional team approach, emphasizing cultural sensitivity, as well as exhibiting compassion and advocacy.

#### Recognizing the social determinants of health

 The TIP interprofessional teams collectively fostered a detailed approach to understanding the patient’s vulnerability in relation to their multimorbidity by paying specific attention to the social determinants of health: *“I do think we do a pretty good job of identifying those social determinants of health and those barriers in our discussions.”* (MD09). Another participant stated: “*Addressing the determinants of health is critical and we end up doing that.”* (DM02).

#### Maximizing a collaborative interprofessional team approach

 The collaborative nature of the team supported sharing ideas about how to overcome some of the barriers experienced by these patients and facilitated creative recommendations.

“This is a skill building exercise for all of us… the people who need our help, like people with complex care needs often are the ones with low SES, low health literacy, not speaking English etc. So maybe it means … let’s get creative.” (DM06)

Having the interprofessional healthcare teams physically together and sharing a collaborative focus on the patient supported system navigation was one approach: “*Because I have got a lot of people around the table, we are able to kind of help them [patients] navigate through the health system a bit better.”* (DM08)*.* Some family physicians who referred to the TIP Program viewed the connections with the interprofessional team as extremely valuable for their vulnerable patients with multimorbidity*.*

“It would be great for that kind of vulnerable patient to have access to all these people, otherwise they might not be able to make those connections. So, if we can make those connections for them that’s great.” (RFP08)

In addition, many participants acknowledged the invaluable role played by team members representing social work and home care: “*As physicians we have some knowledge of these resources but having a knowledgeable social worker is huge in terms of the actual nitty–gritty* of *who’s going to intervene at what time and where.”* (MD09)*.* Through the strong support of home care and social work, access to needed resources for these vulnerable patients with multimorbidity could be expedited: *“They can give them really specific resources that they can literally tap into the same day.”* (HCP04)*.* Social work and home care also helped the team in being realistic: “*So they get us [the team] back on track when the recommendations are not financially feasible.”* (HCP08).

#### Culture sensitivity

 Participants' responses reflected the teams’ cultural sensitivity and commitment to addressing specific cultural issues: “C*ultural beliefs, I think the teams are all very sensitive to that so, we know that if we are dealing with a certain culture and their attitude towards medication or a certain treatment might be different we respect that and work around that.”* (HCP15)*.* When possible, teams made an effort to familiarize themselves with the patients’ cultural background: *“I think when there are different cultures then we try to learn more prior to the session or we just ask the patient when we are in the session.”* (HCP19).

#### Compassion and advocacy

 Participants viewed the patients’ struggles of experiencing multimorbidity and vulnerability through a compassionate lens: “*People have often been minimally engaged for reasons beyond their control and now are really struggling often with difficulties as a result of that.”* (HCP19)*.* Participants acted as advocates for these vulnerable patients with multimorbidity: “*They don’t necessarily demand care, but we advocate this care for them because we see they are in need and if we don’t give it to them they will just keep on returning to the emergency room.”* (MD10).

Overall, participants believed that the Program was specifically useful for vulnerable patients with multimorbidity: *“It opens up doors for the most vulnerable.”* (HCP14)*.* Another participant explained: *“I would say those vulnerable populations are the ones who likely need our help most… it’s exactly what they need. If you do have a lot of bio-psycho-social challenges this is really the way that care would probably be best delivered.”* (DM08)*.* Participants noted how the main issues experienced by patients with multiple chronic conditions often had little to do with the medical comorbidities but rather with barriers outside of the medical model. Consequently, addressing these issues was frequently *“The first step to that patient addressing their chronic illness.”* (DM05). They described the Program as often being the first time patients experienced feeling heard: “*I think a lot of them felt really heard and I have had a patient say directly to me ‘I am so glad you found me”.* (HCP09)*.*

## Discussion

Study findings revealed that participants placed specific attention on a description of patients with multimorbidity and their added vulnerabilities. For example, participants emphasized how these vulnerable patients with multimorbidity frequently struggle with both accessing and then navigating the healthcare system. Similarly, in a study on care for vulnerable patients in deprived areas in Montreal, Canada, physician participants described how the majority of the patients they cared for were living in poverty and had multiple chronic conditions [14]. Other research has noted how this population in addition to challenges in navigating the healthcare system, [33–35] experiences more difficulty in obtaining quality care usually in terms of access and coordination. These issues are exacerbated in immigrant populations who lack familiarity with the local healthcare system [36–38]. 

Our participants expressed that a patient's vulnerability was often compounded by significant mental health issues which in turn could be exacerbated by social determinants of health including poverty, homelessness, and social isolation. These findings build on prior research. A scoping review on mapping the concept of vulnerability related to healthcare disparities [2] also described a model of multi-vulnerability (co-existence of multiple vulnerability factors). The top factors identified were poverty, racial/ethnic minority and chronic physical or mental illness. Our findings are of added value to this review, which had noted the lack of qualitative studies describing the concept of vulnerability in relation to multimorbidity.

Our study participants articulated how the team maximized opportunities for interprofessional collaboration for example; providing a care plan, communicating and sharing physical space, all of which have been previously highlighted in the literature [39–41]. Furthermore the diversity of the team's knowledge and skill sets could facilitate ready access to a variety of community and healthcare resources.

Cultural sensitivity was highlighted by our participants as a core strategy in working with these vulnerable patient populations experiencing multimorbidity. Of note, participants referred to cultural sensitivity versus cultural competence [42]. In a review on interventions that improve cultural competences by Butler et al., 2016 [43] the authors describe that next to provider education and training, changing clinical environments are also key to improving culturally competent care. Our participants actually describe TIP as an intervention that helps providers to better understand the cultural components of clinical encounters with different populations and reduced their own inherent biases through patient-centered interprofessional collaboration. Providers working in the intervention actually improved their ability to provide healthcare services to their target population and assist these patients to competently navigate the broader health system.

Prior research has reported that healthcare providers often face many professional and personal barriers in caring for patients with multimorbidity who are vulnerable, particularly those living in poverty resulting in the provision of suboptimal care [13, 16, 17]. In contrast, our participants explicitly inquired about whether patients were experiencing circumstances that could contribute to their being vulnerable. They approached care of these patients with compassion and a commitment to serve as their advocate. In essence they engaged patients in what some authors describe as relationship-centered discussions about their social determinants of health [14, 39]. Our findings reinforce the importance of being an advocate for patients with multimorbidity as they navigate the healthcare system [44].

### Limitations

All our participants were active team members in the TIP Program and may be biased in their perspectives of the Programs' outcomes. It is challenging to mitigate this risk because in qualitative research participants who have a rich perspective on the topic are required. The experiences of patients with multimorbidity who are vulnerable were not included in this analysis. As a result, we do not know about their perceptions or experiences of being vulnerable. This is a limitation and needs to be a focus of future research.

## Conclusion

This one-hour consultation, outside of usual care, involving allied healthcare professionals (e.g., nursing, social work, pharmacy), physician specialists (e.g., psychiatry, internal medicine) and family physicians, may overcome barriers faced by vulnerable patients with multimorbidity. This Program recognizes and tackles the multiple issues of these patients with multimorbidity who are also vulnerable due to, for example, mental health issues, social isolation, ethnicity, and poverty.

## Data Availability

The datasets used and/or analysed during the current study are available from the corresponding author on reasonable request. Data are not public for reasons of confidentiality.
